# Demographic and Clinical Features of Early-Onset versus Late-Onset Gender Dysphoria Among Adolescent and Young Adult Birth-Assigned Females

**DOI:** 10.1007/s10508-026-03571-6

**Published:** 2026-09-19

**Authors:** Hollie Hammond, Emma Walter, Martin Daly, James S. Morandini, Evelyn Smith

**Affiliations:** 1 School of Psychology, Western Sydney University, Locked Bag 1797, Sydney, Penrith, NSW 2751 Australia; 2 King Street Psychology Clinic, Sydney, Australia

**Keywords:** Gender dysphoria, Transgender, Non-binary, Sexual orientation, Mental health, DSM-5-TR

## Abstract

This study examined differences in demographic and clinical features between early- and late-onset gender dysphoria (GD) in a sample of 138 birth-assigned female adolescents and young adults (AYAs) aged 16–25 years. Participants were recruited through social media and word of mouth and completed an online survey. Onset type was determined using retrospective self-report on adapted DSM-5-TR criteria for childhood GD. Results showed most participants (79%) were classified as late-onset. Late-onset participants were significantly more likely to identify as non-binary, while the early-onset group more frequently endorsed binary identities (e.g., man/male). Early-onset participants reported significantly more childhood gender nonconformity, comorbid diagnoses, and more severe GD and anxiety. Participants classified as late-onset reported limited evidence of childhood gender nonconformity. These findings support the notion that GD is heterogeneous with regard to onset, severity, and comorbidity in AYA birth-assigned females. Further research is needed to investigate these divergent pathways and their clinical implications.

**Supplementary Information:**

The online version contains supplementary material available at https://doi.org/10.1007/s10508-026-03571-6.

## Introduction

Gender dysphoria (GD) describes the distress experienced when one’s gender identity does not align with one’s sex and has garnered increasing attention over the last decade. Research has sought to establish how best to understand GD, particularly regarding adolescents and young adults (AYAs) and to establish best-practice interventions (Becker–Hebly et al., 2021; de Vries & Cohen–Kettenis, 2012; de Vries et al., 2011). These efforts have been complicated by the significant (and seemingly increasing) heterogeneity of GD in adolescent populations (Aitken et al., 2015; Fahrenkrug et al., 2025; Zucker, 2019).

Before moving forward, it is important to address the issue of language. The term birth-assigned sex has a specific history in discussions of newborns with differences/disorders of sex development, where sex classification may not be straightforward on visual inspection of the genitalia (Meyer-Bahlburg, 1998). That broader discourse is beyond the scope of the present paper. This paper uses sex to refer to sex observed at birth, sometimes called birth-assigned or natal sex; consequently birth-assigned females will be referred to as females. Gender is used to refer to gender identity. Sexual orientation will be discussed in relation to sex, unless otherwise specified. Finally, it is important to address the distinction between GD and gender identity, given the widespread conflation of these constructs in mainstream media and academic research. For example, some researchers conflate persistence of gender dysphoria symptoms with persistence of transgender identity (e.g., Temple Newhook et al., 2018). Such scholarship uses the identity category of transgender (where a person identifies with the experience of their gender being different from their sex) interchangeably with the diagnosis or symptoms of GD (distress over the difference between gender and sex). Though there is significant overlap between GD and gender identity, they are not the same thing; a transgender identity is not inherently pathological, and the diagnosis of GD does not automatically entail a transgender identity. Research regarding GD should not adopt a posture of pathologizing transgender identities. This paper concerns itself with transgender identities only insofar as they are adopted by individuals experiencing GD symptoms, because the primary focus of this paper is the demographic and clinical features of AYA females experiencing GD.

### Sexual Orientation and Age of Onset

Sexual orientation and age of onset of GD symptoms have been important for understanding GD in males. Compared with population averages, an overrepresentation of same-sex attraction has consistently been observed among people with GD (Drummond et al., 2008; Zucker, 2017); however, exploring the relationship between GD and orientation has been complicated by issues of operationalization in contemporary literature. For example, some research analyzes sexual orientation using self-identification unmoored from sex (e.g., Turban et al., 2022a, 2022b), whereas others consider identity, behavior, and attraction (e.g., Reisner et al., 2023), and others analyze orientation using a combination of the previous two, establishing scales relative to sex (e.g., Drummond et al., 2008). This represents a particular challenge when studies do not differentiate their results based on sex, as results can become onerous to interpret or compare, if not impossible.

Regarding onset, historically and diagnostically GD has been understood to follow at least two developmental pathways: early- and late-onset (Schneider et al., 2016; Zucker, 2019). Early-onset (EO) typically begins in childhood and is marked by strong gender nonconformity and an association with same-sex attraction in males and females upon sexual maturity (American Psychiatric Association, 2022; Chivers & Bailey, 2000). Although there is debate about the utility of understanding persistence in GD (Temple Newhook et al., 2018; Zucker, 2018), this means that EO children will often be same-sex attracted in adulthood, whether their GD persists or desists (Lawrence, 2010; Ristori & Steensma, 2016; Singh et al., 2021; Steensma et al., 2013). Co-occurrence of same-sex attraction and gender nonconforming behavior, which is observed cross-culturally (Bartlett & Vasey, 2006) and across species (Olvera-Hernández et al., 2015), is thought to both be the outcome of sex-atypical prenatal neurodevelopment (Bailey & Zucker, 1995; Manzouri & Savic, 2019; Rahman & Wilson, 2003; Xu et al., 2021).

Conversely, late-onset (LO) GD in males typically emerges during (or after) puberty (Lawrence, 2010). This LO pathway is characterized by other-sex (i.e. non-homosexual) attraction, absence of pervasive childhood gender nonconformity, and clinically significant GD onsetting after puberty, and is well-documented in males and represents the majority of historical clinical cases discussed in literature (Lawrence, 2010). In some cases, LO males may be aware of gender-related yearnings or wishes even in childhood, and it is only in adolescence or adulthood (i.e., sexual maturity) that these feelings may reach the clinical threshold for GD and motivate outward expression of gender identity and/or some form of transition (Zucker et al., 2016). Whereas many such individuals would not pursue transition until life circumstances allowed in middle age, increasingly this pathway appears to be onsetting in adolescence, perhaps owing to greater acceptance and visibility of transgender identities. A minority of contemporary LO cases may be thought of as etiologically more similar to EO (i.e., pervasive gender nonconformity and same-sex attraction), however with persistent and insistent feminine gender identity not emerging until adolescence or adulthood. In contrast, LO GD in females did not appear in the clinical literature until the 1990s, and has become the dominant clinical presentation over the last 10–15 years (Aitken et al., 2015; Chivers & Bailey, 2000; de Graaf et al., 2018; Kaltiala-Heino et al., 2015).

### Sex and Age of Onset

Contemporary GD literature includes more of a balance of male and female participants (Bauer et al., 2022; Petruzzelli et al., 2025; Topaz et al., 2024). Despite this, much of the existing evidence to support early use of gender-affirming care (GAC) was based on research conducted with mostly male children who demonstrated marked gender nonconformity from early childhood, evidenced clear signs of GD prior to puberty, and did not demonstrate the same prevalence of serious comorbid and preexisting psychopathology observed in contemporary samples. Some exceptions to the male predominance in the earlier gender-affirming care literature exist, though the exceptions pertain to EO females with GD. For example, de Vries et al. (2011) reported slightly more females than males (37 vs. 33), and Smith et al. (2001) reported more females than males in the early-treated group (13 vs. 7) but more males than females in the delayed-treatment group (13 vs. 8); de Vries et al. (2014) also included female patients (for a critical discussion of this literature, see Abbruzzese et al., 2023). Patterns in clinical samples and parent-report convenience samples suggest females experiencing GD may, in contrast, be more likely to experience a later onset of GD, exhibit little childhood gender nonconformity, and demonstrate severe psychopathology preceding the onset of GD (de Rooy et al., 2024; Diaz & Bailey, 2025; Hutchinson et al., 2020; Kaltiala-Heino et al., 2015; Littman, 2018; Zucker, 2019). On these grounds, debate regarding the suitability of GAC for adolescents who do not fit the profile found in earlier literature has increased among researchers and clinicians, notably in response to increased numbers of adolescent females presenting to gender clinics (Aitken et al., 2015; Biggs, 2020; Clayton, 2023; de Graaf et al., 2018; Drobnič Radobuljac et al., 2024; Fahrenkrug et al., 2025; Kaltiala-Heino et al., 2015; Topaz et al., 2024).

There has been considerable controversy regarding how to account for both the observed increase in presentations and the apparent heterogeneity observed among adolescent females experiencing GD (Aitken et al., 2015). Some have attributed the increase in female adolescents presenting to gender clinics to more liberal social attitudes to transgender people. In this view, efforts to destigmatize GD and transgender identities are understood to have removed barriers to “coming out” and seeking GAC, thus more female adolescents are seeking help for GD and adopting transgender identities (Ashley, 2020; Twenge et al., 2025)*.* Others have suggested the disproportionate increase in AYA females suggests the emergence of a new phenomenon, namely a sociogenic pathway to GD with an unknown likelihood of persistence (Diaz & Bailey, 2025; Littman, 2018; Zucker, 2019). In this view, the disconnect between demographic and clinical features in the evidence-base for GAC and that of the contemporary population of adolescents with GD is cause for concern. Finally, others have observed a combination of both positions. On one hand, bullying and abuse of gender nonconforming children and adolescents has decreased while demand for clinical services has increased, as changing social attitudes would predict. On the other hand, mental health has deteriorated during the same time—particularly among females—as a sociogenic phenomenon might predict (Morandini et al., 2022).

Although clinical referrals have come to be predominated by adolescent-onset female cases of GD accompanied by significant comorbid psychopathology (Kaltiala-Heino et al., 2015; Thompson et al., 2022), most research does not distinguish between developmental subtypes (early vs. late). Some recognition of the importance of timing and onset of GD is reflected in the literature, though results are often not reported by sex, and onset operationalization remains heterogeneous (Petruzzelli et al., 2025). One systematic review found that none of the studies included in their analysis (*n* = 38) reported any data on age of onset (Thompson et al., 2022). This means much of the contemporary research either assumes the historical patterns in onset, sexual orientation, and GAC efficacy observed in predominantly male samples will be shared by predominantly female samples (though it is unclear whether this is the case), or it neglects these features entirely. The limits to comparison are exacerbated by heterogeneous approaches to reporting sex, sexual orientation, and onset, further complicating our understanding of GD development in females. Recent attempts to examine onset-related differences between sexes remain limited, for example one clinical study collected data between 2007 and 2011 used retrospective self-report data to classify adult patients as EO or LO, finding onset type was related to GD severity in males (*n* = 178), though they could not report on females due to a small sample size as only twenty of 140 females were categorized as LO (Schneider et al., 2016). In contrast, clinical data collected between 2013 and 2021 made use of similar retrospective self-report methods, finding approximately equal distribution of EO and LO between male and female patients, and differences in dysphoria severity by both sex and onset type (Fahrenkrug et al., 2025). Taken together, these findings underscore the infrequency of retrospective self-report LO females prior to 2015 and highlight outstanding questions in relation to onset type in females.

As for sex-based differences, recent research using age at first clinical presentation as a proxy for onset has found that older-presenting adolescents are more often female, report less childhood nonconformity (Arnoldussen et al., 2023), and demonstrate greater psychological distress than their younger-presenting counterparts (Sorbara et al., 2020) (*n* = 1487 and *n* = 300 respectively). In addition to potential differences in mental health characteristics and dysphoria development in samples based on sex, there may be other clinically relevant distinctions based on sex, including distinct GD typologies within each sex characterized by distinct etiological factors (Koehler et al., 2017; Steensma & Cohen-Kettenis, 2015). This highlights the heterogeneous presentations, measures, and approaches to understanding adolescents with dysphoria and the need for greater attention to age of onset and sex in the assessment of adolescent GD.

### Gender Dysphoria as a Symptom of a Primary Mental Disorder

Limited empirical research has tested the sociogenic hypothesis, with current evidence primarily drawn from two parent-report studies (Diaz & Bailey, 2025; Littman, 2018). According to parents in these studies, the “rapid” development of dysphoria is characterized by female sex, an absence of GD and/or gender nonconformity in childhood, frequent presence of comorbid mental health disorders, and reports of friends who came out as transgender around a similar time (Diaz & Bailey, 2025; Littman, 2018). Some have explained the absence of childhood gender nonconformity by suggesting that while there may have been no *observable* history of GD or gender nonconformity, perhaps females experiencing LO GD may have experienced an internal sense of dysphoria that would not have been evident to parents or clinicians (Ashley, 2020). Thus far, these parent-report studies used divergent methods to assess development and onset of GD, with one using modified criteria based on the *Diagnostic and Statistical Manual of Mental Disorders* (5th ed., text rev.; DSM-5-TR; American Psychiatric Association, 2022) for GD in childhood (Littman, 2018), and the other asking when participants’ children began to show symptoms of GD (Diaz & Bailey, 2025). These parent-report studies gave rise to the primary rapid-onset GD (ROGD) hypothesis that there is a new pathway to developing GD which may be a product of peer influence. They have also given rise to a secondary hypothesis that this rapid form of GD may be a maladaptive mechanism for coping with distress (Littman, 2018).

The hypothesis that ROGD is a maladaptive coping mechanism suggests GD may present as part of a preexisting symptom (e.g., identity diffusion) of a primary underlying mental illness (e.g., borderline personality disorder or autism spectrum disorder), rather than as a stand-alone diagnosis itself, implying a need for greater scrutiny in clarifying and evaluating differential diagnoses in the current diagnostic process. Beyond the parent-report data, the evidence for this hypothesis is drawn from recent scholarship on detransition, such as Vandenbussche et al. (2022), who reported that 70% of their sample (*n* = 237) had come to understand that their GD related to “other issues” (p. 1607). This may be maladaptive as adolescents who interpret their GD symptoms (which are possibly transient) as evidence of being transgender (which is understood as more permanent)—and therefore ego-syntonic—may be motivated to undertake medical interventions which may not be clinically indicated. Some evidence for the potential harm of interventions which may not have been clinically indicated is similarly drawn from the detransition literature. For example more than half of participants in one study of detransitioners (*n* = 20) expressed feelings of grief and regret over undergoing GAC (Pullen Sansfaçon et al., 2023), while others (*n* = 28) felt regret relating to an absence of individualized care and adequately informed consent (MacKinnon et al., 2023). Beyond any issue of grief and regret, there is evidence that adolescents with GD experience psychiatric symptoms and comorbidity which is comparable to other clinical patients without GD, underscoring the possibility that GAC may not be an adequately individualized intervention in a psychiatrically heterogeneous group (Karvonen et al., 2022). If medical intervention can cause harm when not clinically indicated, it is important to improve understanding regarding differential diagnoses and other factors pertinent to mental health and demographic features of AYAs with GD.

Criticism of the ROGD studies has focused on their use of parent-report data (without adolescent self-report data), and the pathologization of transgender identities (Ashley, 2020; Restar, 2020). While transgender identities and GD symptoms are distinct phenomena, it is also clear there is significant overlap between the two. The putative increase of transgender identity is relevant to the ROGD hypotheses only insofar as it is the result of developing psychiatric symptoms (e.g., GD) in parallel to peers, which are then interpreted as containing important information regarding identity. This information regarding identity may then be thought to entail various forms of GAC.

Given these concerns with current literature, the existence of an ROGD pathway remains highly contested. Some researchers have claimed to test the hypothesis empirically, but their methods and interpretations have been challenged (e.g., Bauer et al., 2022; Littman, 2022; Sinai, 2022; for review, see Zucker, 2026). Similarly, the U.S. Transgender Survey dataset (*n* = 27,497) was analyzed by two groups, with one group concluding that a minority of participants “realized” their gender identity late, providing evidence against the ROGD hypothesis (Turban et al., 2023). The other group concluded that an overwhelming majority of the sample could be considered LO, most of whom were also female, in turn concluding that the data provided evidence in favor of the ROGD hypothesis (Sapir et al., 2024).

No study to date has surveyed a non-clinical sample of female AYAs with GD regarding their level of childhood gender nonconformity, onset type, sexual orientation, distress and well-being, dysphoria severity, and comorbidity. This study aims to answer the question of whether onset type is associated with any demographic or clinical differences in a sample of female adolescents with GD. As there is a dearth of research on this question, the intent of this study is primarily exploratory.

## Method

### Participants

Participants were 138 females between the ages of 16 and 25 who indicated they experience GD. Participants were recruited via social media (e.g., Facebook ads, Reddit), and word of mouth as part of a larger study on adolescent GD. The study was open to anyone between the target ages who experienced GD and could read English. To address ethical issues regarding capacity to consent to research among younger adolescents, we included adapted Gillick Competence items drawn from information in the Participant Information Sheet (e.g., What is this survey about?). All participants in this subsample answered all three Gillick multiple choice questions correctly and were understood to be capable of consent. Following consent, participants were asked their age and whether they experienced GD. Anyone who indicated an age under 16 or over 25, or who reported they did not experience GD was screened out. Participants were then asked a range of demographic questions, followed by questions regarding GD, and their mental health.

To ensure data quality, surveys completed in under 5 min were excluded, as this was a priori deemed insufficient time for attentive participation. Further, Qualtrics proprietary bot-detection was enabled, and all responses were manually reviewed for inattentiveness or patterned responding. No responses were identified as suspicious or non-serious and the full data set was retained for analysis. Finally, only participants who completed at least one psychometric measure were included for analysis (i.e., those completing only demographic information were screened out, *n* = 52), yielding the final sample of 138.

### Procedure

Recruitment flyers and links/QR codes were shared via Facebook advertising and on Reddit forums r/asktransgender and r/non-binary with moderator approval, as well as on general research participation forum r/samplesize. Survey information was also shared with email and research contacts. Participants were invited to share the survey with others, should they wish to do so. Participants were recruited between March and April 2024.

A survey instrument consisting of demographic questions, targeted questions related to the research aims, and psychometric measures of psychological phenomena was administered via Qualtrics. Participants took an average of 37 min to complete the survey. Participation was anonymous by default; however, participants could indicate their interest to be contacted in future for follow-up research. The survey instrument will be made available from the corresponding author upon reasonable request.

### Measures

*Demographics*. Participants were asked questions about their age, sex, ethnic/cultural identity, relationship status, current occupation, and perceived support from parents and peers. In addition, participants were asked about their gender identity, choosing all that apply from a list including binary and non-binary options (e.g., non-binary, gender fluid, transmasculine, man/male, agender). Sexual orientation was measured using an adapted version of the Kinsey scale (Kinsey et al., 1998). Here, participants were classified as gynephilic (female-attracted), androphilic (male-attracted), ambiphilic (both-sex attracted), or asexual (no attraction to either sex).

*Development and Onset of Gender Dysphoria*. The survey attempted to understand development and onset of GD in multiple ways. Participants responded to 8 criteria A items adapted from the DSM-5-TR diagnostic criteria of childhood GD. Participants were asked to recall their experiences between ages 3 and 11, indicating whether or not they recalled meeting any of the 8 symptom items. Cronbach’s alpha for these items was 0.83. We summed these items and created a dichotomous variable using the childhood diagnostic cut-off. Per DSM-5-TR childhood criteria, those who reported meeting at least 6 symptoms for a minimum of 6 months were classified into the EO group (i.e., recalled meeting childhood diagnostic criteria). Those who met fewer than 6 symptoms were placed in the LO group (i.e., did not recall meeting childhood diagnostic criteria). Thus, late-onset classification should be understood as not meeting the DSM-5-TR childhood symptom threshold; it does not require complete absence of any childhood gender nonconformity. All subsequent analyses were based on this categorization. We also take the sum of symptoms endorsed to indicate degree of childhood gender nonconformity.

Additionally, participants were asked to indicate the age at which they first began to experience symptoms of GD. Participants were also asked directly whether they identify as something other than cisgender (yes or no) and those who indicated yes were asked at what age they first identified as something other than cisgender. Participants were also asked whether they have come out to others as something other than cisgender, and at what age they first did so.

*Comorbid Psychiatric Diagnoses*. Participants were provided with a checklist of 33 DSM-5-TR diagnoses and asked to indicate which they had received from a mental health professional, if any. For analysis, the number of participants endorsing each diagnosis was compared between groups. The total number of diagnoses was also computed to allow for between-group comparison of diagnostic burden. Comparisons were made between groups on the prevalence of each diagnosis. Finally, diagnoses were also collapsed into DSM-5-TR categories for analysis (e.g., affective disorders) to allow for comparison of the number and type of diagnostic categories between early- and late-onset groups. A dichotomous variable representing any diagnosis at all versus no diagnosis at all was also computed for the purpose of comparing prevalence of any comorbidity.

*Gender Dysphoria Severity*. Dysphoria severity was assessed using the Dysphoria subscale of the Utrecht Gender Dysphoria Scale-Gender Spectrum (UGDS-GS) (McGuire et al., 2020). The UGDS-GS is inclusive of all gender identities and is appropriate for use with adolescents. Participants are given a set of statements such as “I feel unhappy because I have the physical characteristics of my assigned sex” and asked to indicate their level of agreement from 1 (completely disagree) to 5 (completely agree). Scores on items are averaged, and scores closer to 4 on the Dysphoria subscale items are taken to indicate presence of dysphoria. Cronbach’s alpha for this subscale was 0.89.

*Mental Health—Distress*. A general dimension of distress was assessed using the DASS21, a short form measure of the DASS. The DASS is comprised of 3 subscales measuring depression, anxiety, and stress (Henry & Crawford, 2005). To interpret DASS21 scores in line with DASS cut-offs, scores were doubled. Each subscale ranged from 0 to 42. Cronbach’s alpha for the depression, anxiety, and stress subscales was 0.89, 0.87, 0.83 respectively.

*Mental Health—Well-being*. Well-being was assessed using the Warwick-Edinburgh Mental Well-being Scale (WEMWBS), a 14-item measure of mental well-being centered on detecting positive aspects of mental health in the previous two weeks (Tennant et al., 2007). Participants indicate their agreement with statements such as “I have been feeling relaxed and I have been feeling optimistic about the future.” Items are summed, with higher scores indicating higher well-being. Cronbach’s alpha for this scale was 0.89.

### Analysis

Data were analyzed using SPSS Version 29.0.2.0. We first conducted two one-factor MANOVAs on conceptually related outcomes to control for multiple comparisons and test for differences by onset type. The first included GD symptom and identity outcomes (number of childhood symptoms endorsed, age of GD onset, age at first non-cis identity, age of “coming out” as non-cis, years of non-cis identification), with onset type entered as the factor (EO vs. LO). The second included mental health outcomes (DASS-21 subscales, WEMWBS, and the UGDS-DS Dysphoria subscale), and onset type was again entered as the factor. We undertook missing data analysis on all measures and found missing data from one participant (0.7%) on the DASS21 measure only. We conducted a post-hoc power analysis using G*Power (version 3.1.9.7) to evaluate adequacy of power for the analysis including DASS21 data, given sample size, effect size, and significance level. The analysis revealed that the test achieved a high power of 0.961, suggesting sufficient statistical power. The MANOVAs were used to provide a multivariate omnibus test of group differences. Where the multivariate test was significant, we inspected the individual dependent variables with one-way ANOVAs. Some variables contained valid missingness (for example, age at coming out only included participants who had previously indicated that they were out to others), and so we used per-variable ANOVAs to preserve the maximum sample for each outcome. Cohen’s d has been provided for clinical interpretability.

Due to the proportional nature of the data, differences in the prevalence of diagnoses were tested by comparing onset types using chi-square analysis. Independent t-tests were used to test for differences in the total number of comorbidities between EO and LO groups.

Owing to overlap on distribution of GD symptom onset between EO and LO groups, we conducted post-hoc analyses to test whether there were more appropriate methods for categorizing onset. We dichotomized the single item “Please indicate the age at which you first began to experience gender dysphoria” (i.e., GD symptom onset) using three different cut-off points in line with key events in longitudinal clinical literature. We tested key outcomes using the following cut-offs: at/before age 6 versus after age 6; at/before age 8 versus after age 8; and before age 10 versus at/after age 10. The first two represent key ages at which EO participants were first transitioned in Olson et al. (2022), and the last represents an approximation of symptom onset before and after female pubertal onset (Eckert-Lind et al., 2020). Differences on key outcomes were comparable to the main analysis, and none of these alternate methods appeared clearly superior to our original classification approach. For those who reported receiving a formal diagnosis of GD, we also considered testing EO/LO classification using the single item “Age at which you received your GD diagnosis”; however, no participants reported receiving a diagnosis prior to age 12. To test whether a categorical approach was justified at all, we tested two continuous variables (age at which GD symptoms began vs. sum of childhood GD criteria endorsed) using a simultaneous regression model on key mental health outcomes. The results follow the same pattern as our main analysis, with continuous variables yielding no additional explanatory power over our categorical approach (see Supplementary Table 5).

## Results

### Sample Characteristics and Onset Type

A total of 138 participants aged between 16 and 25 (M age = 19.57 years, SD = 2.68) participated in the study. Using DSM-5-TR criteria for childhood GD, 29 participants were classified as EO, and 109 participants were classified as LO (21% and 79%, respectively). Gender identity was diverse, with transmasculine the most frequently endorsed gender identity across EO and LO groups (58.6% and 54.1%, respectively). Male/man was the next most frequently endorsed gender identity in the EO group (40.4%), with smaller percentages identifying across a range of other identities (Table 1). LO participants were significantly more likely to identify as non-binary than EO participants, *χ*^2^(1, *N* = 138) = 3.83, *p* = .050. Most participants were single (67.4%) and born in Europe (71.7%). A minority of participants in each group reported a formal diagnosis of GD (Table 1). Over 80% of participants across both groups reported having trans or gender diverse or gender dysphoric friends whom they felt were between somewhat and very good sources of support. Using a modified Kinsey classification, over half the participants in the early-onset (51.7%) and the late-onset (57.8%) groups were female-attracted, either partially (ambiphilic) or entirely (gynephilic).

**Table 1:** Demographic characteristics

	Early-onset^a^		Late-onset^a^	
	M (SD)	%	M (SD)	%
Current age (in years)	19.03 (2.73)		19.72 (2.66)	
*Country of birth*				
Australia		3.4%		3.7%
North America		6.9%		7.3%
Central and Southern America		0.0%		7.3%
Asia		0.0%		1.8%
Europe		75.9%		70.6%
Other		13.8%		9.2%
*Average household income*				
Well above regional average		3.4%		2.8%
Above regional average		20.7%		17.4%
Regional average		34.5%		56.0%
Below regional average		34.5%		15.6%
Well below regional average		6.9%		8.3%
*Occupation*				
Attending high school		48.3%		38.5%
Attending university or college		34.5%		42.2%
Completing a trade qualification		0.0%		2.8%
Employed full-time		6.9%		8.3%
Employed part-time		10.3%		16.5%
Not employed or studying		10.3%		11.0%
*Relationship status*				
Single		75.9%		65.1%
In a relationship		24.1%		34.9%
*Sexual orientation (modified Kinsey)*				
Gynephilic		27.6%		23.9%
Ambiphilic		24.1%		33.9%
Androphilic		24.1%		19.3%
Asexual		24.1%		22.9%
*Formal diagnosis of GD*				
Yes		44.8%		31.2%
No		55.2%		68.8%
Age of formal GD diagnosis	15.92 (1.80)		18.24 (2.82)	
Number of DSM-5-TR childhood symptoms endorsed	6.72 (0.80)		1.79 (1.76)	
*Do you have TGD or GD friends?*				
Yes		86.2%		83.5%
No		13.8%		16.5%
Perceived support from transgender/GNC/GD friends^b^	4.80 (0.50)		4.67 (0.68)	
Number of transgender/GNC/GD friends prior to GD realization^c^	6.11 (5.35)		4.89 (4.49)	
*Is your primary parent or carer aware you experience GD?*				
Yes		62.1%		55.0%
No		37.9%		45.0%
Perceived support from parents^d^	3.35 (1.39)		3.65 (1.29)	
*Gender identity*				
Male/man		41.4%		28.4%
Female		0.0%		2.8%
Transfeminine		0.0%		0.9%
Transmasculine		58.6%		54.1%
Non-binary		20.7%		40.4%
Genderfluid		10.3%		7.3%
Questioning		3.4%		4.6%
Agender		3.4%		10.1%
Other		6.9%		5.5%

^a^ Calculated from number of items endorsed from DSM-5-TR childhood GD criteria

^b^ Average support rating from 1, very unsupportive, through to 5, very supportive

^c^ Sum of number of online and in person friends reported prior to participants realizing they experience GD

^d^ Average perceived support rating from 1 (very unsupportive) to 5 (very supportive)

### Gender Dysphoria Development and Onset

The MANOVA indicated a significant and large multivariate effect of onset type across GD development variables, Pillai’s *V* = .649, *F*(4, 125) = 57.84, *p* < .001. We then inspected ANOVAs for each onset variable, finding a significant and large effect of onset type on number of DSM-5-TR childhood symptoms endorsed, with EO participants reporting significantly more childhood symptoms (*M* = 6.72, *SD* = 0.78) than LO participants (*M* = 1.79, *SD* = 1.76), *F*(1, 136) = 214.37, *p* < .001, *η*^2^ = 0.612, Cohen’s *d* = 3.05, 95% CI [2.51, 3.60]. EO and LO participants differed on other measures of GD onset and identity development, with EO individuals beginning to identify with a non-cisgender identity earlier (*M* age = 12.68 years, *SD* = 2.76) compared to late-onset individuals (*M* age = 15.01 years, *SD* = 2.74), *F*(1, 134) = 16.01, *p* < .001, *η*^2^ = 0.107, *d* = − 0.84, 95% CI [− 0.86, − 0.03] (Table 2). A similar significant effect of onset type was demonstrated on the age at which participants first “came out” to others with a non-cisgender identity, with EO participants coming out earlier (*M* age = 13.86 years, *SD* = 2.40) than LO participants (*M* age = 16.16 years, *SD* = 2.75), *F*(1, 130) = 16.58, *p* < .001, *η*^2^ = 0.113, *d* = − 0.85, 95% CI [− 1.28, − 0.43] (Table 2). Number of participants and degrees of freedom reflect valid missingness (e.g., participants not out to others excluded from the analysis of “coming out”).

**Table 2:** ANOVA comparing GD onset variables and timing of identity changes

	Early-onset			Late-onset			*p*	*F* (df)	*η*^2^
	*n*	M	SD	*n*	M	SD			
Number of DSM-5-TR childhood symptoms endorsed	29	6.72	0.78	109	1.79	1.76	< .001*	214.37 (1, 136)	.612
Age of GD symptom onset	29	9.41	3.02	109	11.17	4.11	.034*	4.59 (1, 136)	.033
Age at first non-cis identity	28^b^	12.68	2.76	108	15.01	2.74	< .001*	16.01 (1, 134)	.107
Age of “coming out” as non-cis	29	13.86	2.40	103	16.16	2.75	< .001*	16.58 (1, 130)	.113
Years since “coming out”^a^	29	5.17	3.66	103	3.67	2.11	.042*	8.03 (1, 130)	.058

^a^ Years since coming out calculated by subtracting the age at which participants indicated they “came out” with a non-cisgender identity from their age at survey completion. ^b^ One participant reported they did not adopt a non-cisgender identity, but also reports being “out” to others; participant appears to identify as (and be out to others as) male, but does not identify as transgender. The absolute range for the number of DSM-5-TR childhood symptoms endorsed is 0–8

The overwhelming majority of EO (91.7%) and LO (96.6%) participants had taken social steps (such as changing pronouns or clothing style) to manage dysphoria symptoms, which did not differ significantly between groups (Supplementary Table 2). Of the EO group, 22% and of the LO group 24% (*p* = .808) reported managing GD with hormonal steps. Of the EO group, 8.3% and of the LO group 10.3% (*p* = .723) reported managing GD with surgical steps (Supplementary Table 2).

The DSM-5-TR criteria for childhood dysphoria most likely to be endorsed by the EO group were the items pertaining to dressing preferences and preferences for cross-sex roles in make-believe play (Supplementary Table 3). Strong preference for cross-sex clothing, followed by a strong preference for cross-sex toys were most often endorsed by the LO group, though these remained in the minority. The items pertaining to internal feelings (dislike of one’s sexual anatomy and desire for attributes of the other sex) were endorsed least often by the LO group (Supplementary Table 3).

### Comorbidities

The majority of both EO (75.9%) and LO (56.9%) onset participants reported the presence of at least one diagnosed comorbidity, with no significant difference between onset types (Supplementary Table 4). Chi-square tests revealed a small effect of onset in relation to individual diagnoses. EO participants reported significantly more attention deficit/hyperactivity disorder, generalized anxiety disorder, and panic disorder than LO participants. EO participants also reported more narcissistic personality disorder than LO participants, however the LO group contained a zero cell and therefore the comparison should be interpreted cautiously (Table 3). EO individuals reported a significantly higher number of diagnosed comorbidities compared with LO individuals, Welch’s *t*(33.36) = 2.30, *p* = .028. The group difference was 1.48 diagnoses, 95% CI [0.17, 2.80], corresponding to a medium effect, *d* = .64, 95% CI [0.022, 1.06]; see Table 4. At the category level, EO participants also reported more obsessive-compulsive and related diagnoses (20.7%) compared to the late-onset group (8.3%, *χ*^2^(1, *N* = 138) = 3.984, *p* = .046, *V* = .177; (Supplementary Table 4).

**Table 3:** Chi square results for diagnoses found to differ significantly between groups

	Early-onset % (*n* = 29)	Late-onset % (*n* = 109)	*p*	*χ*^2^ (df)	Effect size (Cramer’s *V*)
ADHD	27.6%	11.9%	.037	4.35(1)	.178
GAD	41.4%	22.0%	.035	4.45(1)	.220
PD	20.7%	5.5%	.010	6.65(1)	.180
NPD^a^	6.9%	0.0%	.006	7.63(1)	.235

*ADHD* is attention deficit hyperactivity disorder, *GAD* is generalized anxiety disorder, *PD* is panic disorder, *NPD* is narcissistic personality disorder. Table shows percentage of participants indicating they have received this diagnosis. All Cramer’s V values reflect a small effect size. All other diagnoses did not differ significantly between groups. See Supplementary Table 4 for full prevalence of comorbidities. aDue to zero cell count in the LO group, this result should be interpreted with caution

**Table 4:** Independent samples *t*-test of number of formal diagnoses by onset type

Variable	*n*	M	SD	*P*
*Number of formal diagnoses*				
Early-onset	29	3.07	3.33	0.028
Late-onset	109	1.59	1.96	

unequal variances indicated, Levene’s *F*(1, 136) = 11.98, *p* < .001; inferential tests reported in-text use Welch’s *t*

### Gender Dysphoria Severity, Distress, and Well-being

There was a significant multivariate effect of onset type across the combined outcomes, Pillai’s *V* = .150, *F*(5, 131) = 4.61, *p* < .001; we then inspected follow-up ANOVAs. Scores on the UGDS-GS showed that EO participants experienced significantly higher dysphoria severity (*M* = 4.46, *SD* = 0.60) compared to LO individuals (*M* = 3.96, *SD* = 0.64; *F*(1, 136) = 15.51, *p* < .001, *ηp*^2^ = .102; *d* = 0.82, 95% CI [0.40, 1.24], Table 5). Additionally, anxiety levels, as measured by the DASS were significantly higher among EO participants (*M* = 20.50, *SD* = 12.18) than LO participants (*M* = 14.75, *SD* = 10.30; *F*(1, 135) = 6.43, *p* = .012, *ηp*^2^ = .045, *d* = 0.53, 95% CI [0.15, 0.96]; Table 5). Both EO and LO groups ranged from moderate to extremely severe on each of the three DASS subscales, such that neither group scored below the moderate range on any distress subscale. No significant differences were observed on measures of well-being, depression, or stress.

**Table 5:** MANOVA univariate results of onset differences using M scores on measures of dysphoria, distress, and well-being

	Early-onset		Late-onset		*p*	*F* (df)	*η*^2^
	M	SD	M	SD			
UGDS-GS							
Dysphoria	4.48	0.60	3.96	0.64	< .001*	15.51 (1136)	.102
Distress (DASS)^a^							
Depression	22.64	12.64	20.51	11.26	0.386		
Anxiety	20.50	12.18	14.75	10.30	0.012*	6.43 (1,135)	.045
Stress	22.50	11.50	20.17	9.57	0.272		
Well-being (WEMWBS)	39.83	11.15	38.95	9.04	0.661		

UGDS range = 1–5; DASS subscales range = 0–42; WEMWBS range = 14–70. ^a^ *n* for EO group is 29, except for DASS subscales where it is 28; *n* is 109 for LO across all variables

## Discussion

This study aimed to answer the question of whether onset type is associated with any demographic or clinical differences in a sample of female adolescents with GD. Using DSM-5-TR criteria of childhood dysphoria, we divided our sample into an EO group (21%) who retrospectively reported having met childhood dysphoria criteria, and an LO group (79%) who did not. The LO group indicated minimal evidence of childhood gender nonconformity (e.g., cross-sex playmate, clothing, and toy preferences) and minimal or no cross-sex desire in childhood. Further, we found a significant association between LO classification and a non-binary identity, which to our knowledge has not been reported previously, and a high prevalence of same-sex attraction. In terms of peers, EO and LO participants both reported having a handful of transgender or gender diverse friends prior to realizing they experienced GD (approximately 6 and 5, respectively), with no significant difference between groups. Regarding mental health, the EO participants reported significantly more formally diagnosed comorbid disorders, and significantly more severe GD, while the LO participants reported significantly less anxiety.

### Onset and Identity

We found using the DSM-5-TR cut-off for GD in childhood was associated with a significant difference in number of symptoms endorsed between the groups, such that the EO group reported significantly more symptoms in childhood, and the LO group reported a minimal history of symptoms of GD. This may seem obvious, as of course those who met the diagnostic cut-off and those who did not would be different, however this is not necessarily the case. Although EO participants would, by definition, meet a minimum of 6 symptoms, there is no reason to assume the LO participants would not also report 3, 4, or even 5 symptoms on average. Childhood symptoms of GD did not need to be significantly different. In fact, if it were the case that adolescent-onset GD in females was primarily experienced by those with a history of gender nonconformity or private symptoms of dysphoria as Ashley (2020) has argued, we would expect to see a higher number of childhood symptoms endorsed in the LO group, particularly on items related to private, unobservable desires. Accordingly, our use of late-onset denotes not meeting the childhood symptom threshold rather than the complete absence of any childhood gender nonconformity. As it stands, LO participants themselves report fewer than two symptoms in childhood, with the onset of their GD appearing to truly begin during puberty, a finding similar to that of the older presenters in Arnoldussen et al. (2023). LO participants most endorsed DSM-5-TR criteria relating to gendered behavior and roles rather than cross-sex desires or anatomical dysphoria. This suggests there may be a link with a social form of dysphoria, and raises the possibility that the GD experienced by this group may not be solely characterized as dysphoria in relation to participants’ sexed bodies. One study of detransitioners suggested the overwhelming majority of their participants reported both social and body dysphoria (Vandenbussche, 2022), providing some indirect support for the possibility that GD in females who begin social transition in adolescence may be, at least in part, experiencing a social form of dysphoria.

Further, the finding that almost 80% of our sample aligns with this LO pathway is a near complete inversion of clinical findings collected between 2007–2011 (Schneider et al., 2016). However, this finding shares some similarities with those of Sapir et al. (2024) who reported 75% of their USTS-15 sample was classified as “late realization”, and Littman et al. (2024) who found 83% of previously transgender-identified participants retrospectively reported not having met criteria for GD in childhood per the DSM-5. This is noteworthy, as it suggests our sample shares important features with a much larger non-clinical sample, and a sample of detransitioners. This commonality with detransitioners raises questions about the transience of GD symptoms in adolescent females which should be explored further.

Regarding GD symptom timing, the EO group in our sample reported first developing GD symptoms at a little over nine years old (approximately prior to puberty), significantly different from our LO group who reported GD symptoms beginning at just over 11 years old (approximately early in puberty). These trajectories continue in a staggered manner, with EO participants first coming out as non-cisgender at almost fourteen years old, and LO participants 2 years later at approximately 16 years old. There are elements of our EO group which do not appear to align with characteristics of EO cohort studies showing children with GD had presented to the clinic by approximately age 6 and had transitioned and endorsed (predominantly) binary transgender identities by approximately age 8. This contrasts with our sample, with EO participants privately and publicly adopting non-cisgender identities between approximately 12.5 and 14 years old. Our EO sample diverged in other key ways from clinical cohort studies, for example reporting an older age of symptom onset and—for those reporting a formal GD diagnoses—of diagnosis (Olson et al., 2022). Despite meeting our retrospective cut-off criteria for EO classification, our participants do not necessarily align with prospective clinical EO characteristics. Our EO group may instead represent a pseudo-EO pathway which may more accurately be described as late childhood/early adolescent onset. The timing of coming out for our EO participants took place on average in early 2019, and for our LO participants, in later 2020. Timing may relate to social isolation and time to reflect during the pandemic, with some evidence that females may have been more likely to report changes in gender identity during this time (Bianchini et al., 2024). Additionally, we also found a significant association between LO participants and reporting a non-binary identity, which has not been reported in previous research. This finding was unexpected, as there has been no indication in the literature of a relationship between gender identity and onset of GD symptoms. One meta-analysis has found that non-binary identities are associated with worse mental health than transgender or cisgender identities (Klinger et al., 2024), while another recent study found non-binary identities are one variable which may be associated with increased odds of stopping or reversing gender-affirming medical treatment (MacKinnon et al., 2024). Therefore, mental health and GD persistence among non-binary AYAs should be a focus of future research.

### Sexual Orientation and Peer Relations

Our sample also indicates a high prevalence of same-sex attraction, though these did not differ significantly between groups. According to Rahman et al. (2020), 90.7% of females in the general population are considered heterosexual using Kinsey classification, however in our sample that figure was just 24.1% for EO and 19.3% for LO participants. Despite an unusually high prevalence of same-sex attraction, no relationship was found between onset type and sexual orientation, suggesting our sample may diverge from male GD trends which have associated the EO pathway with same-sex attraction, and the LO pathway with non-homosexual attraction (Lawrence, 2010). Our results suggest that patterns in sexual orientation which have been observed in males with GD may not be shared by females with GD symptoms, warranting further exploration. It also remains possible that sexual orientation differs among EO persisters and desisters, although available data do not clearly document whether EO desisters are more likely to be heterosexual than early-onset persisters; future longitudinal research should examine this question directly.

Our participants reported approximately 2–3 in-person transgender friends prior to realizing their own experience of dysphoria, though there was no difference between EO or LO groups on total number of friends, online friends, or in-person friends. While this finding is in line with parent-reports in Diaz and Bailey (2025), who reported an average number of 2.4 friends prior to coming out, it is challenging to interpret. As the sociogenic hypothesis of GD is highly controversial, few studies ask questions about friendships in relation to symptom onset or identity realization, so comparison becomes difficult. Our full sample overlaps with that of Diaz and Bailey (2025) in this regard; however, if our LO group overlapped with the hypothesized sociogenic (ROGD) cohort, we would expect to see LO participants reporting more trans and gender diverse friends prior to GD realization. In terms of peer support, both groups reported their trans and gender diverse friends as being somewhat to very supportive, with no significant differences between the two.

### Clinical Features

The EO group reported significantly more comorbid diagnoses, approximately doubling the LO group. In terms of individual diagnoses, EO participants reported significantly more attention deficit/hyperactivity disorder, generalized anxiety disorder, panic disorder, and narcissistic personality disorder. It is possible the EO group experiences increased mental health difficulties relative to the LO group, which could be due to stigmatizing experiences during childhood and adolescence due to being gender nonconforming (Wittlin et al., 2023; Xu et al., 2026). For example, there may be a pattern of initial GD symptomatology, with other comorbidities developing in response to negative environmental reactions to early gender nonconformity, as recently suggested by Xu et al., (2026). Conversely, it is also possible that the EO group—by virtue of experiencing symptoms at a younger age—has had more contact with mental health professionals for support with their dysphoria and is therefore more likely to receive formal diagnoses. Simultaneously—though lower overall—the LO group was not without distress and comorbidity, and here it is possible there is an inverse pattern such that preexisting comorbidities may lead to the experience of GD symptoms. It is important that future research attempts to more carefully understand the temporal order of events, particularly in clinical samples where triangulation of onset variables may be more feasible. The raw prevalence of at least one other mental health disorder in our LO group (56.9%) was somewhat closer than our EO group (75.9%) to Littman’s (2018) parent-report value (62.5%). This suggests that LO adolescents themselves report similarities with Littman’s parent-report sample on comorbidities, and this overlap offers some support for the ROGD hypothesis. In terms of dysphoria severity, the EO females did, in fact, demonstrate higher symptom severity than our LO participants, extending previous findings and suggesting onset may be a clinically important variable (American Psychiatric Association, 2022; Schneider, 2016).

On measures of depression, stress, and well-being, EO and LO participants did not differ significantly, though similarly to Bauer et al. (2022) the LO group reported significantly less anxiety than the EO group (i.e., moderate rather than severe). One explanation for this would be similar to the explanation for higher comorbidity in EO participants: that more severe anxiety symptoms are driven by greater stigma during childhood and adolescence (Wittlin et al., 2023). Regarding the hypothesis of maladaptive coping, one difficulty lies in establishing relative distress between EO and LO participants. For example, while parents have characterized their adolescent-onset children as highly distressed (e.g., Littman, 2018), this cannot tell us anything about whether LO distress is more or less severe than EO distress. This means parents might classify their children who score in or above the moderate range on measures of distress (as found in our LO sample) as very distressed, though it cannot imply more (or less) distress than the EO group. On this basis, our finding that anxiety is higher in our EO group does not allow us to rule out the possibility that LO GD represents a pathway where distress precedes GD. While we cannot rule out this possibility, we are cautious in offering a strong conclusion on the issue of directionality, given we do not have robust data on whether GD or distress/other diagnoses came first. This, perhaps, reiterates the importance of multiple sources of reporting when it comes to classifying childhood and adolescent behavior, as well as longitudinal research capable of monitoring mental health over time.

### Strengths and Implications

The findings of the present study are supported by several strengths. We have addressed a number of methodological and operational limitations of previous research by recruiting adolescents directly via general social media channels related to GD and being transgender rather than from online communities who are skeptical of GD and/or of being transgender. Further, we used valid psychometrics with reliability data (e.g., Cronbach’s alpha), and asked salient questions directly to AYAs, rather than using parent-report. Additionally, we used adapted DSM-5-TR criteria to separate participants into EO and LO groups which meant our cut-off point for categorization was not restricted to features of our own sample as it would be if, for example, we used histograms of age of symptom onset data, though we explored these alternatives in our post-hoc analyses.

Using onset type to compare our sample, we found many similarities between our LO participants and the parent-report samples described in Littman (2018) and Diaz and Bailey (2025), as well as with characteristics of the sample in the USTS-15 reanalysis by Sapir et al. (2024). Further, we found some overlap with a sample of previously transgender-identified adolescents who endorsed the rapid pathway as describing their experience (Littman et al., 2024), and a similar lack of childhood gender nonconformity to that found by Arnoldussen et al. (2023). This research also suggests contemporary cohorts of female adolescents with EO GD may diverge from clinical cohorts (Olson et al., 2022) in important and clinically relevant ways which deserve further exploration.

The present findings have possible implications for clinical practice with gender dysphoric females. First, EO participants were found to be at greater risk of mental health comorbidities, as well as higher anxiety and more intense dysphoria. Understanding factors underlying this elevated risk would be critical to reducing psychiatric morbidity in this subgroup. Our findings suggest clinicians should consider taking careful histories of symptom onset, as onset may be informative regarding the type and number of comorbidities and other mental health symptoms associated with dysphoria development in females. Further, our findings suggest there may be an association between GD onset and gender identity, which may have implications for clinical interventions. Finally, our results suggest that a minority (21%) of female adolescents who come out as transgender during puberty are likely to report a childhood history of GD symptoms or gender nonconformity.

### Limitations

Ideally, our study and those like it would follow a very large sample of participants longitudinally from early childhood to adulthood, collecting data on relevant variables such as gender identity, gender nonconformity, gender expression, sexual orientation, and mental health at multiple ages. Such a study should bolster self-report data with additional reports, such as those from parents, teachers, or therapists. This design would allow for more accurate understanding of the developmental features of dysphoria and the relationship between onset and mental health and could offer some protection against threats of recall bias or post-event information effects. As such a design was not feasible within our project, our study has several limitations. We had a small and uneven convenience sample which was reliant on self-report, and a minority who reported clinical engagement and a formal diagnosis. While this design has allowed us to explore our questions in a non-clinical sample of adolescents, it is necessarily not clear whether our results may apply to clinical samples of adolescents with GD.

On the shortcomings of retrospective self-report, others have raised the possibility of recall bias with regard to gendered behavior in childhood (Bailey & Zucker, 1995), and those limitations cannot be discounted here. One recent study suggested that neuroticism leads to overestimation of negative affect in adolescent retrospective self-report (Schmidt et al., 2023). All our participants were at least moderate or higher on measures of distress, and our EO participants most of all. Measures of distress are understood to be highly correlated with neuroticism, which may confound measures of anxiety, stress, and depression (Jylhä & Isometsä, 2006; McCrae, 1990). Therefore, it is possible our most distressed participants may inadvertently bias their own recollection toward recalling increased symptom severity in childhood. This could result in those most distressed in the present being classified EO, which may not accurately reflect their developmental pathway.

One way to improve understanding of the relationship between recalled childhood gender nonconformity and distress in the present may be to control for personality variables. Further, this research also highlights the possibility that recalling childhood behavior from a single self-report source has limitations, and future research may wish to employ multiple reports to triangulate results, should they endeavor to use retrospective age of onset classification.

Finally, our cross-sectional, observational method necessarily prevents us from establishing any causality when it comes to development of dysphoria, and our results should not be interpreted as making causal claims. For example, our method cannot clearly answer the question of whether early experiences of GD may be causal to other mental health challenges, or whether other mental health challenges may be causal to later GD.

### Future Directions

Prospective longitudinal research should seek to more confidently establish age of onset of GD symptoms to corroborate whether EO individuals do in fact demonstrate more elevated distress than their LO counterparts. Longitudinal clinical research comparing groups based on age at presentation or self-reported recall of age of onset (in adolescent presentations) may continue to face challenges regarding recall bias. To guard against the possibility of neuroticism confounding measures of distress and even of onset classification, future research should also consider controlling for personality factors. Including questions on gender identity and expression within new or existing social network analytic research settings or large cohort studies may represent one viable avenue of investigation. Alternatively, research could begin to see if there are alternate ways to operationalize group differences in females with GD which are less subject to the limitations of retrospective self-report, for example by examining personality traits or comorbidities for meaningful patterns.

### Conclusion

The present study contributes to our understanding of an under-researched cohort and highlights the importance of contemporary research into GD given recent shifts in clinical presentation demographics. Most adolescent females experiencing GD report little gender nonconformity in childhood. LO and EO groups are associated with heterogeneity on several demographic and clinical variables, though differences in distress and well-being were minimal. Further research into GD onset in contemporary female adolescents is warranted.

## Supplementary Information

Below is the link to the electronic supplementary material.

Supplementary file1 (DOCX 21 KB)

## Data Availability

The data cannot be made publicly available due to conditions of ethics approval. Participants did not consent to have their responses shared publicly in any fashion, or with anyone outside of the named research team. The survey instrument as it pertains to this paper will be made available from the corresponding author upon reasonable request.

## Declarations

### Code Availability

No custom code was used in this study. All analyses were conducted using IBM SPSS version 29.0.2.0 graphical user interface.
