# Supplementary material for: Demographic and Clinical Features of Early-Onset versus Late-Onset Gender Dysphoria Among Adolescent and Young Adult Birth-Assigned Females
Source: Arch Sex Behav. 2026 Sep 19;55(7):2947–62. doi: 10.1007/s10508-026-03571-6 (PMC13748164; doi:10.1007/s10508-026-03571-6)
Supplement: Supplementary file 1 [file 10508_2026_3571_MOESM1_ESM.docx]

Supplementary Tables

**Supplementary Table 1**

*Development and onset of gender dysphoria for full sample*

| Variable | N | M (SD) | % |  |  |
| --- | --- | --- | --- | --- | --- |
| Age of symptom onset | 138 | 10.80 (3.96) |  |  |  |
| Using non-cisgender identity |  |  |  |  |  |
| Yes | 136 |  | 98.60 |  |  |
| No | 2 |  | 1.40 |  |  |
| Age of non-cisgender identity | 136 | 14.53 (2.90) |  |  |  |
| Age of “coming out” as non-cis | 132 | 15.65 (2.83) |  |  |  |
| Formal diagnosis of GD |  |  |  |  |  |
| Yes | 47 |  | 34.10 |  |  |
| No | 91 |  | 65.90 |  |  |
| Received formal GD diagnosis from |  |  |  |  |  |
| Psychiatrist | 20 |  | 42.60 |  |  |
| Psychologist | 17 |  | 36.20 |  |  |
| Family doctor | 2 |  | 4.30 |  |  |
| Other | 8 |  | 17.00 |  |  |
| Number of DSM-5-TR childhood symptoms endorsed |  | 2.83 (2.58) |  |  |  |
| Met DSM-5 cut-off for GD diagnosis in childhood |  |  |  |  |  |
| Yes | 29 |  | 21.00 |  |  |
| No | 109 |  | 79.00 |  |  |

**Supplementary Table 2**

*Steps taken to manage gender dysphoria*

|  | Early-onset (*n* = 29)  % | Late-onset (*n* = 109)  % | *p* |
| --- | --- | --- | --- |
| Social | 91.7 | 96.6 | .375 |
| Hormonal | 22.0 | 24.1 | .808 |
| Surgical | 8.3 | 10.3 | .723 |
| Psychotherapeutic | 39.4 | 31.0 | .476 |
| No steps taken | 6.9 | 3.7 | .449 |

**Supplementary Table 3**

*Frequency of childhood symptom endorsement by onset type*

| DSM-5-TR Gender Dysphoria in Childhood item | Early-onset | Late-onset |
| --- | --- | --- |
| A strong desire to be the other (natal) sex | 86.2% | 21.1% |
| A strong preference for dressing as the other (natal) sex | 93.1% | 33.0% |
| A strong preference for cross-sex roles in make believe play | 93.1% | 20.0% |
| A strong preference for toys, games, or activities that are stereotypically used by the other (natal) sex | 79.3% | 32.1% |
| A strong preference for playmates of the other sex | 86.2% | 19.3% |
| A strong rejection of typically masculine (if male) and typically feminine (if female) toys, games, and activities | 75.9% | 22.9% |
| A strong dislike of your sexual anatomy | 82.8% | 14.7% |
| A strong desire for the physical attributes that match the other natal sex | 75.9% | 15.6% |

**Supplementary Table 4**

*Prevalence of diagnoses and suspected diagnoses*

| Variable | Early-onset  %  (n = 29) | Late-onset  %  (n = 109) |
| --- | --- | --- |
| Diagnosed mental health disorders |  |  |
| At least one comorbid diagnosis | 75.9 | 56.9 |
| No comorbid diagnosis | 24.1 | 43.1 |
| Type of comorbidity^a^ |  |  |
| Affective disorder | 37.9 | 27.8 |
| Anxiety disorder | 48.3 | 34.3 |
| Neurodevelopmental disorder | 37.9 | 23.1 |
| Obsessive disorder^b^ | 20.7 | 8.3 |
| Trauma disorder | 13.8 | 8.3 |
| Personality disorder | 3.4 | 5.6 |
| Eating disorder | 13.8 | 4.6 |
| Dissociative disorder | 6.9 | 1.9 |
| Conduct disorder | 0.0 | 0.9 |
| Other disorder | 8.7 | 10.2 |
| No comorbid disorder | 10.1 | 42.6 |
| Specific Diagnoses |  |  |
| Generalised Anxiety Disorder^c^ | 41.4 | 22.0 |
| Major Depressive Disorder | 37.9 | 26.6 |
| Social Anxiety Disorder | 34.5 | 20.2 |
| Attention Deficit Hyperactivity Disorder^c^ | 27.6 | 11.9 |
| No Disorder | 24.1 | 43.1 |
| Autism Spectrum Disorder | 20.7 | 18.3 |
| Panic Disorder^c^ | 20.7 | 5.5 |
| Obsessive Compulsive Disorder | 13.8 | 5.5 |
| Post-Traumatic Stress Disorder | 10.3 | 4.6 |
| Specific Phobia | 10.3 | 2.8 |
| Body Dysmorphic Disorder | 10.3 | 2.8 |
| Anorexia Nervosa | 10.3 | 4.6 |
| Any Dissociative Disorder | 6.9 | 1.8 |
| Narcissistic Personality Disorder^c^ | 6.9 | 0.0 |
| Bipolar Disorder | 3.4 | 1.8 |
| Specific Learning Disorder | 3.4 | 3.7 |
| Language Disorder | 3.4 | 0.0 |
| Adjustment Disorder | 3.4 | 4.6 |
| Bulimia Nervosa | 3.4 | 0.0 |
| Substance Use Disorder | 3.4 | 0.0 |
| Avoidant Personality Disorder | 3.4 | 0.9 |
| Borderline Personality Disorder | 3.4 | 2.8 |
| Antisocial Personality Disorder | 3.4 | 1.8 |
| Motor Disorder | 0.0 | 0.9 |
| Intellectual Disability | 0.0 | 0.9 |
| Tic Disorder or Tourette’s Disorder | 0.0 | 0.0 |
| Oppositional Defiance Disorder | 0.0 | 0.9 |
| Conduct Disorder | 0.0 | 0.0 |
| Dysthymia | 0.0 | 0.9 |
| Schizophrenia or other Psychotic Disorder | 0.0 | 0.0 |
| Dependent Personality Disorder | 0.0 | 0.9 |
| Obsessive Personality Disorder | 0.0 | 0.0 |
| Histrionic Personality Disorder | 0.0 | 0.0 |
| Suspected diagnoses |  |  |
| At least one diagnosis suspected | 86.2 | 89.0 |
| No diagnoses suspected | 13.8 | 10.1 |

^a^ Type of comorbidity is showing percent of cases, e.g., % of participants who indicated an affective disorder. Total does not equal 100% as participants could indicate multiple disorders.

^b^For obsessive disorders, Pearson χ²(1, N = 138) = 3.984, p = 0.046.

^c^For chi square results, please see Table 3 *Chi square results for diagnoses found to differ significantly between groups* (in text).

**Supplementary Table 5**

*Results of simultaneous regression using standardized continuous measures of age of onset and sum of childhood GD symptoms endorsed*

| Outcome | *n* | R² | Adj. R² | F (2, df2) | *p* (model) | β Age of onset | *p* | β GD childhood symptom sum | *p* |
| --- | --- | --- | --- | --- | --- | --- | --- | --- | --- |
| DASS Anxiety | 137 | 0.048 | 0.034 | 3.360 (2, 134) | **.038** | -0.138 | .113 | 0.142 | .104 |
| DASS Depression | 137 | 0.015 | 0.000 | 1.018 (2, 134) | .364 | 0.016 | .855 | 0.125 | .158 |
| DASS Stress | 137 | 0.008 | -0.007 | 0.533 (2, 134) | .588 | -0.081 | .358 | 0.022 | .804 |
| WEMWBS (wellbeing) | 138 | 0.012 | -0.003 | 0.804 (2, 135) | .450 | -0.109 | .217 | -0.004 | .962 |
| UGDS Dysphoria | 138 | 0.225 | 0.213 | 19.547 (2, 135) | **<.001** | **-0.366** | **<.001** | **0.225** | **.005** |
| Total professional diagnoses | 127 | 0.067 | 0.053 | 4.863 (2, 135) | **.009** | -0.082 | .339 | **0.227** | **.009** |

Note. Standardized βs are reported to compare predictors directly. All models are post-hoc and unadjusted; interpret cautiously.
